# Radiomics-Based Pretherapeutic Prediction of Non-response to Neoadjuvant Therapy in Locally Advanced Rectal Cancer

**DOI:** 10.1245/s10434-019-07300-3

**Published:** 2019-03-18

**Authors:** Xuezhi Zhou, Yongju Yi, Zhenyu Liu, Wuteng Cao, Bingjia Lai, Kai Sun, Longfei Li, Zhiyang Zhou, Yanqiu Feng, Jie Tian

**Affiliations:** 1 Engineering Research Center of Molecular and Neuro Imaging of Ministry of Education, School of Life Science and Technology, Xidian University, Xi’an, Shaanxi China; 2 Guangdong Provincial Key Laboratory of Medical Image Processing, School of Biomedical Engineering, Southern Medical University, Guangzhou, China; 3 CAS Key Laboratory of Molecular Imaging, Institute of Automation, Chinese Academy of Science, Beijing, China; 4 Department of Radiology, The Sixth Affiliated Hospital, Sun Yat-Sen University, Guangzhou, China; 5 Department of Radiology, Sun Yat-Sen Memorial Hospital, Sun Yat-Sen University, Guangzhou, China; 6 Collaborative Innovation Center for Internet Healthcare, Zhengzhou University, Zhengzhou, Henan China; 7 University of Chinese Academy of Science, Beijing, China; 8 Beijing Advanced Innovation Center for Big Data-Based Precision Medicine, Beihang University, Beijing, China

## Abstract

**Objective:**

The aim of this study was to investigate whether pretherapeutic, multiparametric magnetic resonance imaging (MRI) radiomic features can be used for predicting non-response to neoadjuvant therapy in patients with locally advanced rectal cancer (LARC).

**Methods:**

We retrospectively enrolled 425 patients with LARC [allocated in a 3:1 ratio to a primary (*n* = 318) or validation (*n* = 107) cohort] who received neoadjuvant therapy before surgery. All patients underwent T1-weighted, T2-weighted, diffusion-weighted, and contrast-enhanced T1-weighted MRI scans before receiving neoadjuvant therapy. We extracted 2424 radiomic features from the pretherapeutic, multiparametric MR images of each patient. The Wilcoxon rank-sum test, Spearman correlation analysis, and least absolute shrinkage and selection operator regression were successively performed for feature selection, whereupon a multiparametric MRI-based radiomic model was established by means of multivariate logistic regression analysis. This feature selection and multivariate logistic regression analysis was also performed on all single-modality MRI data to establish four single-modality radiomic models. The performance of the five radiomic models was evaluated by receiver operating characteristic (ROC) curve analysis in both cohorts.

**Results:**

The multiparametric, MRI-based radiomic model based on 16 features showed good predictive performance in both the primary (*p* < 0.01) and validation (*p* < 0.05) cohorts, and performed better than all single-modality models. The area under the ROC curve of this multiparametric MRI-based radiomic model achieved a score of 0.822 (95% CI 0.752–0.891).

**Conclusions:**

We demonstrated that pretherapeutic, multiparametric MRI radiomic features have potential in predicting non-response to neoadjuvant therapy in patients with LARC.

**Electronic supplementary material:**

The online version of this article (10.1245/s10434-019-07300-3) contains supplementary material, which is available to authorized users.

The standard treatment for patients with locally advanced rectal cancer (LARC) is neoadjuvant chemoradiotherapy followed by total mesorectal excision.^1^ Many studies have demonstrated that the response to neoadjuvant therapy affects prognosis, and that, in particular, pathologic complete response (pCR) has a favorable effect on outcome.^2^–^4^

However, a sizeable proportion of patients show non-response to neoadjuvant therapy. These non-responders can be defined as those who have no tumor regression changes, based on resection specimens, after neoadjuvant therapy. A number of studies have reported that approximately 7% of LARC patients showed non-response to preoperative chemoradiotherapy and more than 20% developed grade 3–4 toxic effects such as diarrhea, nausea, hematological infection, and fever.^5^–^7^ These non-responders may benefit little from neoadjuvant therapy, but still experience the related toxicity. More importantly, tumor progression may occur in a portion of these patients during therapy. Thus, accurately predicting non-responders before administering neoadjuvant therapy is important for devising a personalized treatment plan, which includes avoiding overtreatment and choosing alternative treatments in a timely manner. However, there is currently no robust method for accurately stratifying patients into non-response and response groups, other than by pathologic evaluation after completing neoadjuvant therapy.

Due to the heterogeneity of tumors,^8^ accurately predicting a non-response to neoadjuvant therapy remains challenging. Numerous studies have sought molecular biomarkers for early identification of good and poor responders to neoadjuvant therapy in patients with LARC, but no robust predictive factors have been identified.^9^–^12^

Magnetic resonance imaging (MRI) is the most commonly used diagnostic imaging approach for rectal tumors. Pretherapeutic MRI features, such as tumor volume, tumor height, depth of tumor penetration, and absence of extramural venous invasion, have been associated with a better response to therapy;^13^–^15^ however, these visual features have a poor ability to accurately predict non-response for administering therapy.

Radiomics, a novel approach to medical image analysis, can extract high-throughput quantitative features from images, and thus provides a wealth of information that cannot be assessed visually but is associated with tumoral heterogeneity.^16^ Therefore, tumor characteristics can be better understood by analyzing radiomic features. Radiomics has facilitated progress in precise diagnosis,^17^,^18^ prediction of lymph node metastasis,^19^,^20^ and survival analysis.^21^,^22^ Previous radiomic studies focused on therapeutic responses in LARC patients were mainly based on combining pre- and post-therapeutic imaging data or images obtained during neoadjuvant therapy.^23^–^25^ Most of these studies focused on single-modality imaging, such as dynamic contrast-enhanced MRI,^26^ diffusion-weighted MRI,^27^ T2-weighted MRI,^28^ and fluorodeoxyglucose positron emission tomography,^29^ which may have inherent limitations in reflecting tumor biology. A recent study with a small sample size demonstrated that features derived from multiparametric MRI performed better than that derived from single-modality MRI in terms of predicting good responders from poor responders, but the study lacked independent validation.^30^

Based on the initial success of MRI-based radiomics in assessing pCR, we hypothesized that pretherapeutic MRI-based radiomics would have potential in predicting non-responders to neoadjuvant therapy, and that multiparametric MRI would be more effective than single-modality MRI in this task. Therefore, in this study we investigated whether a pretherapeutic, multiparametric MRI-based radiomic model could predict non-responders to neoadjuvant therapy, as integrating a radiomic model and clinical practice may facilitate a flexible therapeutic schedule prior to commencing neoadjuvant therapy.

## Materials and Methods

### Patients

This retrospective single-center study was approved by the Ethics Committee of the Sixth Affiliated Hospital, Sun Yat-sen University. The need for informed patient consent was waived due to the retrospective nature of this study. Overall, 425 patients with biopsy-proven LARC receiving pretherapeutic, multiparametric MRI examination and standard treatment between November 2012 and May 2017 at this hospital were enrolled; 248 of these patients underwent 5-fluorouracil, leucovorin, and oxaliplatin treatment, and 177 underwent 5-fluorouracil and concurrent radiation treatment (total dose 46.0–50 Gy). The multiparametric MRI consisted of T1-weighted fast spin echo imaging (T1w), T2-weighted fast spin echo imaging (T2w), diffusion-weighted imaging (DWI), and contrast-enhanced T1-weighted fast spin echo imaging (CE-T1w). Two gastrointestinal radiologists with 10 and 30 years’ experience reassessed the rectal tumor in MRI without medical record information, and obtained consistent staging results, including clinical T stage (cT stage) and clinical N stage (cN stage). Other clinical information, including sex, age, and carcinoembryonic antigen (CEA; cutoff ≥ 5 ng/ml, < 5 ng/ml) blood level^31^ was completely recorded. Appendix 1 describes the recruitment of patients.

Pathologic response after neoadjuvant therapy was reassessed based on resection specimens by two pathologists with 10 and 20 years’ experience in consensus. Pathologic grading of primary tumor regression was performed according to the four-tier American Joint Committee on Cancer (AJCC) tumor regression grade (TRG) system.^32^ The four TRG groups are: TRG 0, no residual tumor cells; TRG 1, single tumor cell or small group of tumor cells; TRG 2, residual cancer with desmoplastic response; and TRG 3, minimal evidence of tumor response. According to the AJCC TRG system, patients were categorized into two groups: non-responders (TRG 3) and responders (TRG 0–2). Patients were then randomly divided into two independent cohorts: a primary cohort used for modeling (proportion = 3/4; TRG 3: *n* = 39, TRG 0–2: *n* = 279) and a validation cohort used for testing (proportion = 1/4; TRG 3: *n* = 13, TRG 0–2: *n* = 94). The clinical characteristics of patients in the primary and validation cohorts are summarized in Table 1.

**Table 1:** Clinical characteristics of patients in the primary and validation cohorts

Characteristic	Primary cohort		*p* value	Validation cohort		*p* value
	TRG 3	TRG 0–2		TRG 3	TRG 0–2	
Age, years	50.31 ± 13.89	53.98 ± 12.39	0.089	57.46 ± 8.99	54.06 ± 11.11	0.294
Sex			0.194			0.509
Male	31	191		11	69	
Female	8	88		2	25	
CEA			0.384			0.387
Positive	18	107		4	42	
Negative	21	172		9	52	
cT stage			1			0.744
T2	3	22		0	4	
T3	29	203		11	71	
T4	7	54		2	19	
cN stage			0.573			0.196
N0	6	62		4	21	
N1	18	108		3	47	
N2	15	109		6	26	

Continuous data are expressed as mean ± SD. *p* values for categorical variables, such as sex and CEA, were from the Fisher’s exact test analysis. *p* values for categorical variables, such as sex and CEA, were from the Fisher’s exact test analysis. *p* values for categorical variables, such as cT stage and cN stage, were from the Pearson’s Chi square test analysis. *p* values for continuous variables were from the independent samples *t* test analysis

*TRG* tumor regression grade*, CEA* carcinoembryonic antigen, *cT stage* clinical T stage, *cN stage* clinical N stage

### Image Data Acquisition and Radiomic Signature Construction

Appendix 2 describes the pretherapeutic, multiparametric MRI acquisition; Appendix 3 describes details of tumor masking and radiomic feature extraction; and Appendix 4 describes details of the feature selection method and radiomic signature construction. It should be noted that we built a multiparametric MRI-based radiomic (MPR) model and four single-modality models via the same workflow on the primary cohort only.

### Evaluation and Comparison of Different Prediction Models

Receiver operating characteristic (ROC) curve analysis was conducted in both cohorts to evaluate the predictive ability of radiomic signatures. The Delong test was performed to estimate whether the difference between two arbitrary ROC curves was statistically significant.

Univariate and multivariate logistic regression analyses were conducted to select the most useful predictive clinical variables (Wald test *p*-values < 0.05). A multivariate logistic prediction model was then built by combining the MPR signature and the selected clinical variables; this was termed the CMPR model.

Calibration curves accompanied by the Hosmer–Lemeshow goodness-of-fit (GOF) test were plotted to assess the consistency between the estimated probability and the actual rate of TRG 3; *p* values > 0.05 indicate a good fit of the model.^33^ The classification accuracy, sensitivity, and specificity, according to the Youden index cut-off,^34^ were calculated to quantize the discrimination ability of the prediction models in both cohorts.

## Results

### Demographic and Clinical Characteristics

Detailed information regarding study participants is presented in Table 1 and Appendix Table A3. In this study, we enrolled 425 LARC patients who underwent standard neoadjuvant therapy, including 52 (12.23%) non-responders (42 males, 10 females) and 373 (87.77%) responders (260 males, 113 females). There were no significant differences in clinical variables between cohorts.

### Feature Selection and Radiomic Signature Construction

**Fig. 1:**
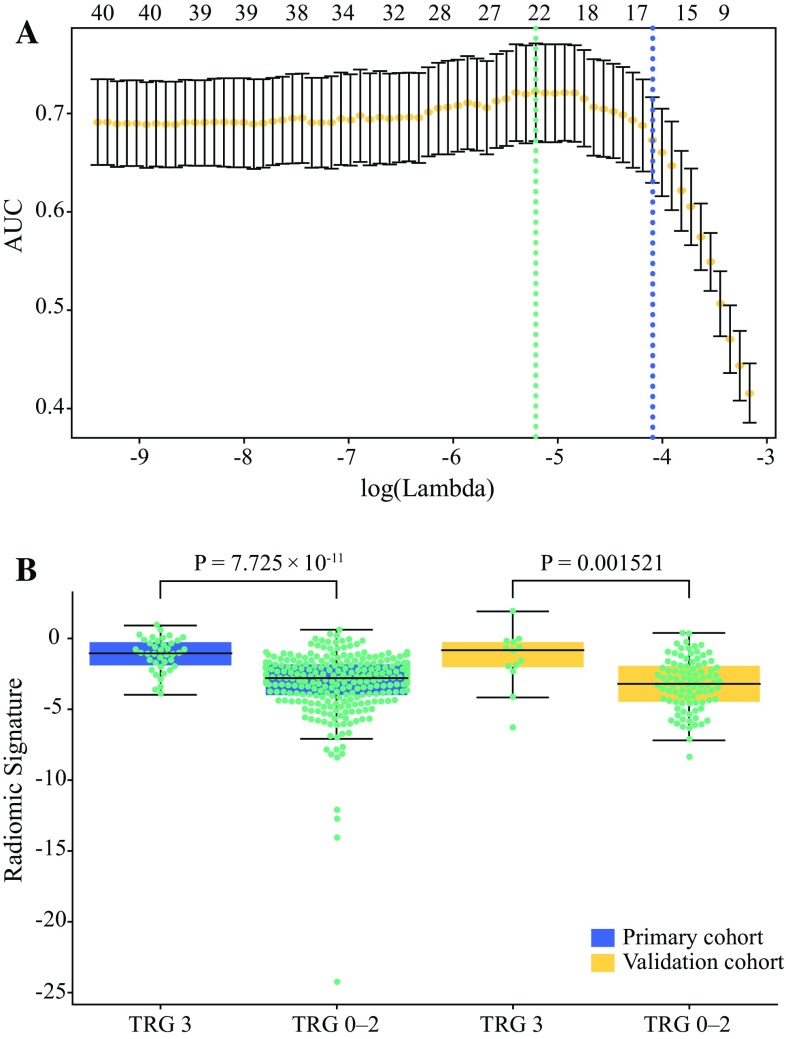
Feature selection using LASSO and distribution of the MPR signature. **a** The ‘Lambda’ parameter was chosen via 1 standard error of the minimum criterion, as marked by the blue dotted vertical line. **b** Distribution of the MPR signature in both cohorts. *LASSO* least absolute shrinkage and selection operator, *MRI* magnetic resonance imaging, *MPR* multiparametric MRI-based radiomic, *TRG* tumor regression grade, *AUC* area under the curve

Sixteen features were selected for constructing the MPR model. In both cohorts, the MPR signature was significantly higher in the TRG 3 group than in the TRG 0–2 group, as shown in Fig. 1. Single-modality radiomic models, including the apparent diffusion coefficient (ADC; seven selected features), T1w (five selected features), CE-T1w (three selected features), and T2w (three selected features) models, were also constructed.

### Evaluation and Comparison of Different Prediction Models

**Fig. 2:**
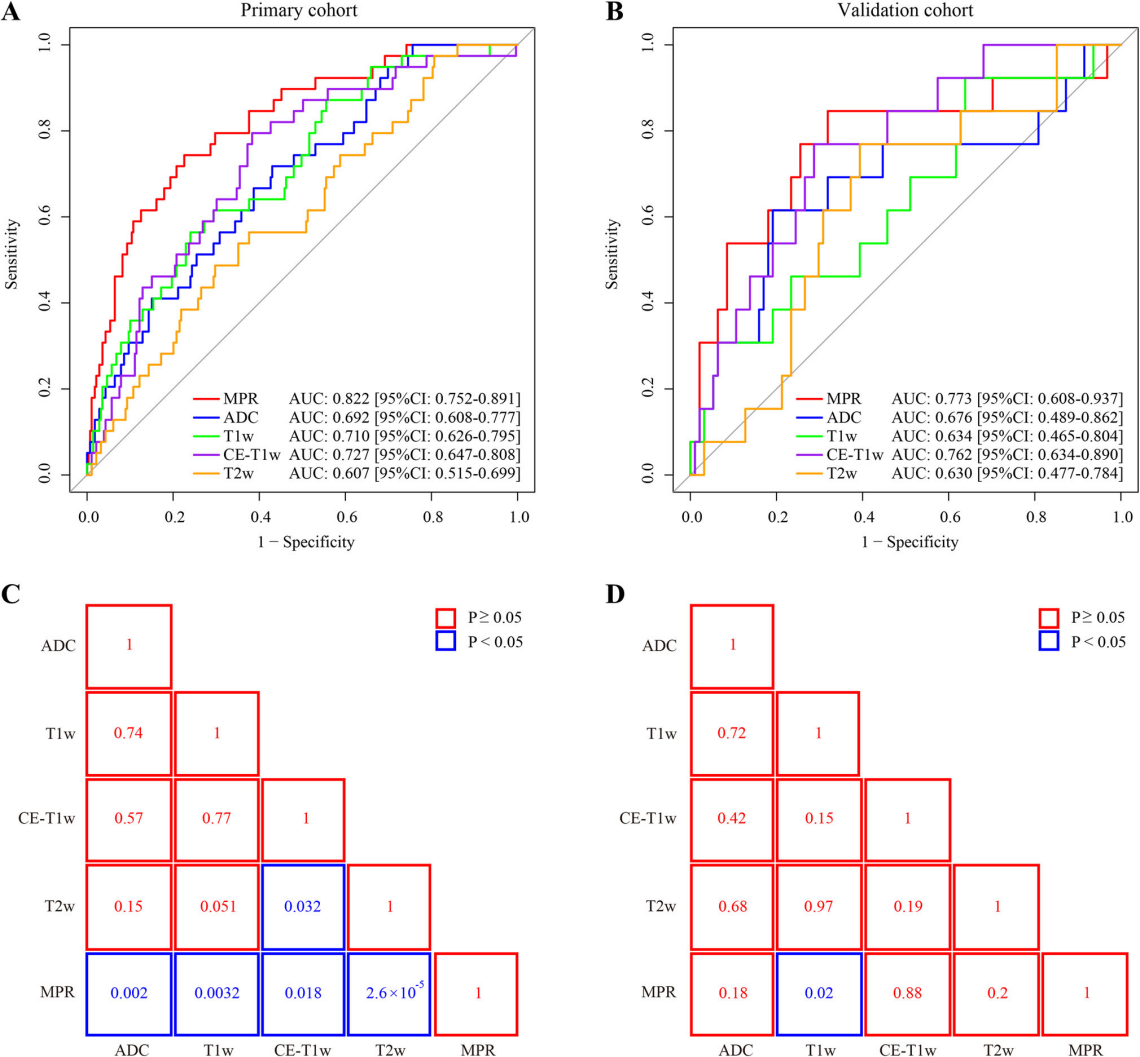
Performance comparison of the MPR and single-modality models. **a**, **b** ROC curves. **c**, **d** Delong test *p*-values. *ROC* receiver operating characteristic, *AUC* area under the curve, *MPR* multiparametric magnetic resonance imaging-based radiomic model, *ADC* apparent diffusion coefficient, *T1w* T1-weighted, *CE*-*T1w* contrast-enhanced T1-weighted, *T2w* T2-weighted*, CI* confidence interval

The MPR model yielded the highest area under the curve (AUC) in both cohorts, and was statistically significantly higher than other single-modality models (*p* < 0.05) in the primary cohort, as shown in Fig. 2.

In the multivariate analysis, both age [odds ratio (OR) 0.57, *p* = 0.0071] and MPR signature (OR 17.78, *p* < 0.001) were significantly associated with non-response, as shown in Table 2. Thus, the CMPR model was built using logistic regression, combining age and the MPR signature in the primary cohort.

**Table 2:** Univariate and multivariate logistic regression analyses for clinical characteristics and MPR signature

Parameter	Univariate			Multivariate		
	*p* value	OR	95% CI	*p* value	OR	95% CI
Sex	0.1645	1.78	0.78–4.04	0.8063	1.21	0.45–2.81
Age	0.0903	0.74	0.52–1.05	**0.0055**	0.55	0.36–0.84
CEA	0.3515	1.38	0.70–2.70	0.4292	1.37	0.86–1.10
cT stage	0.4768	0.63	0.17–2.27	0.2003	0.41	0.63–3.01
cN stage	0.6303	1.24	0.50–3.05	0.6935	0.80	0.27–2.38
MPR signature	**< 0.0001**	14.51	5.82–36.13	**< 0.0001**	17.78	6.73–46.99

The *p* value was from the Wald test analysis. Bold values indicate *p* < 0.05

*OR* odds ratio*, CI* confidence interval, *MPR* multiparametric magnetic resonance imaging-based radiomic model*, CEA* carcinoembryonic antigen, *cT stage* clinical T stage, *cN stage* clinical N stage

**Fig. 3:**
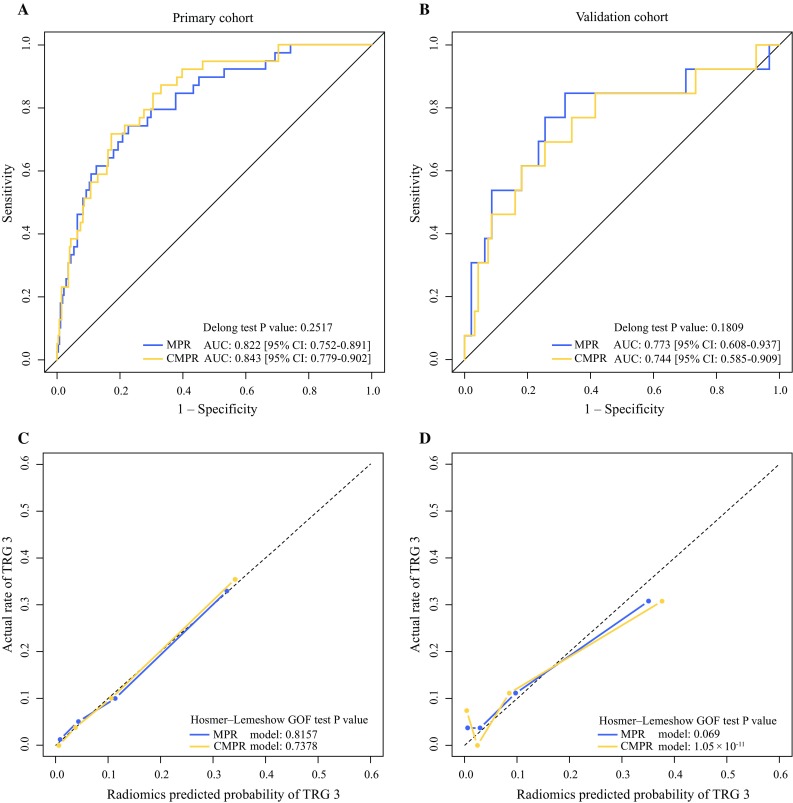
Performance comparison of the MPR and CMPR models. **a**, **b** ROC curves. **c**, **d** Calibration curves. *ROC* receiver operating characteristic, *AUC* area under the curve, *MPR* multiparametric magnetic resonance imaging-based radiomic model, *CMPR* combined MPR signature and age model, *TRG* tumor regression grade, *CI* confidence interval, *GOF* goodness-of-fit

In both cohorts, the CMPR model performed similarly as the MPR model (Delong test *p* values > 0.05) in the ROC analysis. However, the calibration curve generated from the CMPR model did not show good consistency between prediction and actual observation in the validation cohort (Hosmer–Lemeshow GOF test *p* value < 0.05), as shown in Fig. 3. The AUC, accuracy, sensitivity, and specificity of the MPR and CMPR models, according to the Youden index cut-off, are listed in Appendix Table A4.

## Discussion

Our study demonstrated that pretherapeutic, multiparametric MRI radiomic features have great potential in predicting non-responders to neoadjuvant therapy in patients with LARC. Based on the 16 most predictive features, as listed in Appendix Table A5, we established the MPR model by multivariate logistic regression analysis. In an independent validation cohort, the MPR model showed good predictive performance (AUC 0.773).

For radiomic feature extraction, we mainly used the Laplacian of Gaussian (LoG) filter for image preprocessing. An LoG filter can smooth images through different parameter scale settings, which may help to decrease the influence of noise. It can also enhance textural details, which will help to increase the efficiency of capturing phenotypic features that map to tumoral heterogeneity. Because of these advantages, LoG filters have been wildly used in radiomic studies, mostly using CT images.^19^,^35^,^36^ In an early MRI-based radiomic study, an LoG filter was used to extract features for predicting pCR in rectal cancer patients,^37^ while, in the present study, we extracted LoG features on five scales, in addition to the features from the original images. We then selected 16 features and linearly constructed an MPR signature by logistic regression analysis. In the MPR model, there was an apparent difference between the number of features generated from LoG filters and that from the original images. Given that the LoG filter features accounted for a higher proportion of the features employed in the MPR model (10/16) than the original image features, it appears that LoG features may be more suitable than original image features for predicting a non-response. Nevertheless, original image features remain indispensable to the MPR model because the odds ratio of the feature GLCM_entropy_135 generated from the original image was the largest among all the features employed (OR 3.2808, *p* = 0.0059).

In the present study, we also investigated differences in the performance of the MPR and single-modality models. The CE-T1w model was the best-performing single-modality model in both the primary and validation cohorts. Compared with other imaging modalities, CE-T1w images enhanced the contrast of the tumor area, making the texture distribution clearer, which may have helped to improve the predictive ability of the textural feature. The MPR model performed significantly better than the CE-T1w model (*p* = 0.01775) in the primary cohort, implying that each modality has limitations in presenting tumor characteristics. Fusing multimodal information could facilitate a more comprehensive description of tumor characteristics, and could undoubtedly help to establish a more precise prediction model.

Some rectal cancer studies have shown that incorporating clinical variables into the radiomics model can improve predictive performance.^19^,^38^ Hence, we attempted to combine the MPR signature with a clinically associated variable (age) to establish the CMPR model. However, compared with the MPR model, the CMPR model did not improve the predictive ability in the validation cohort. More specifically, the MPR and CMPR models achieved the same predictive accuracy (accuracy = 76.64%), and, compared with the MPR model, the specificity of the CMPR model increased by only 0.97%, while the sensitivity of the CMPR model decreased by 7.69%. Predictive robustness evaluation also showed that the predictive performance of the two models was similar. Therefore, in the present study, we deemed that the MPR model was more suitable.

At present, our understanding of factors influencing the effect of chemoradiotherapy is extremely poor. Several factors, such as apoptosis, proliferation, hypoxia, and cell cycle, have been investigated in this respect.^39^,^40^ Gene mutations in the related pathways have been shown to cause resistance to chemoradiation. With the help of radiomics and increasing medical imaging data, it may be possible to discover a relationship between medical images and non-response. In future, mapping the predictive image features to various molecules will help to discover major biomarkers related to resistance to chemoradiation in a more efficient and economical way.

There are some limitations to this study. First, the proportion of non-responders was small in the cohort as a whole, and, second, this was a single-center study. Repeatability and reproducibility of radiomic features are mainly affected by two factors: segmentation and image acquisition protocol.^41^ In our study, the region of interest was manually delineated by two experienced gastrointestinal MRI radiologists in consensus, resulting in a more accurate segmentation and decreasing the influence derived from interobserver variability. In addition, all these patients were scanned by the same MRI machine with fixed scanning parameters, avoiding the potential impact of a heterogeneous acquisition protocol. In spite of this, and considering the large heterogeneity of data from different centers, the model should be validated using a multicenter dataset before being applied to other institutions. Third, additional clinical information should be collected in future to investigate whether the CMPR model could perform better than the MPR model.

## Conclusions

This study showed that pretherapeutic, multiparametric MRI radiomic features have potential in predicting non-response to neoadjuvant therapy prior to commencing this therapy. In addition, multiparametric MRI was more effective than single-modality MRI in this prediction task. This multiparametric MRI-based radiomic model may help doctors make more appropriate treatment plans for patients with LARC before they receive neoadjuvant therapy.

## Electronic supplementary material

Below is the link to the electronic supplementary material.

Supplementary material 1 (DOCX 861 kb)
