# Supplementary material for: Radiomics-Based Pretherapeutic Prediction of Non-response to Neoadjuvant Therapy in Locally Advanced Rectal Cancer
Source: Ann Surg Oncol. 2019 Mar 18;26(6):1676–84. doi: 10.1245/s10434-019-07300-3 (PMC6510882; doi:10.1245/s10434-019-07300-3)
Supplement: Supplementary file 1 — Supplementary material 1 (DOCX 861 kb) [file 10434_2019_7300_MOESM1_ESM.docx]

**Appendix 1: Inclusion and exclusion criteria**

Inclusion criteria were as follows: (i) biopsy-proven LARC; (ii) complete neoadjuvant therapy; (iii) underwent pre-therapeutic multi-parametric MRI including T1-weighted fast spin-echo imaging (T1w), T2 weighted fast spin-echo imaging (T2w), diffusion-weighted imaging (DWI), and contrast-enhanced T1-weighted fast spin-echo imaging (CE-T1w); (iv) surgery was performed after neoadjuvant therapy, after which pathologic examination had been done; (v) clinical information, including sex, age, clinical T stage (cT-stage), clinical N stage (cN-stage) and CEA (cutoff: ≥ 5 ng/ml, < 5 ng/ml) blood level was completely recorded. Exclusion criteria included the following: (i) lack of multi-parametric MRI data; (ii) insufficient MRI quality due to bowel peristalsis-related artifacts; (iii) lack of clinical information. The recruitment of patients is depicted in **Appendix Fig. A1**.

**Appendix 2: parameter settings of multi-parametric MRI**

Before initiation of neoadjuvant therapy, all patients in this single institution were scanned with 1.5 Tesla MR (Optima MR 360, GE Medical Systems, USA) using an eight-element body array coil with fixed image protocols. The All patients underwent a series of 4 magnetic resonance (MR) scanning sequences, including DWI (2 *b*-values including 0 and 800 s/mm^2^) , T1w, CE-T1w, and T2w. The technical MRI parameters of are listed in **Appendix Table A1**.

**Appendix 3: Tumor masking and radiomic feature extraction**

**Tumor masking**

One gastrointestinal radiologist with 10 years of experience examined the MR images and extracted the region of interest (ROI) by manually outlining the tumor margin, using itk-SNAP software ([www.itksnap.org](http://www.itksnap.org)), on an axial slice. Then, the segmentation was examined by another gastrointestinal radiologist with 30 years of experience. Discrepancy was resolved by discussion and consensus. For each patient, radiologists delineated the ROI only on the slice that showed the largest cross-sectional tumor area on each imaging modality. ROIs on DWI were delineated at a *b* value of 800 s/mm^2^, and were then copied to the corresponding apparent diffusion coefficient (ADC) maps. **Appendix Fig. A2** shows an example of tumor masking.

**Radiomic feature extraction**

The radiomic features extracted are listed in **Appendix Table A2**. In total, 606 features were extracted from each of T1w, T2w, CE-T1w images, and apparent diffusion coefficient (ADC) maps. These features could be divided into 3 categories, including first-order statistics, GLCM textural features, and Laplacian of Gaussian (LoG) filtration features. A total of 2424 features were extracted from each patient’s MR images and were linearly normalized into a range [-1, 1]. The radiomic feature extraction was conducted using in-house software written in MATLAB (MathWorks, Inc., Natick, MA, USA). All specific calculation formulas could be easily obtained in previous studies [^1^](#_ENREF_1)^,^[^2^](#_ENREF_2), thus, here we only provide the names of features we extracted as follows:

(1) **Image filtration**

A Laplacian of Gaussian (LoG) spatial band-pass filter was used to derive image features at different spatial scales by turning the filter parameter between 1.0 and 3.0 (1.0, 1.5, 2.0, 2.5 and 3.0). The $\text{LoG}$ filter distribution is given by

$\text{LoG}\left(\text{x},\text{y}\right)=\frac{-1}{\pi\sigma^{4}}\left(1-\frac{x^{2}+y^{2}}{2\sigma^{2}}\right)e^{-\frac{x^{2}+y^{2}}{2\sigma^{2}}}$,

of which$\text{x and y}$denote the spatial coordinates of the pixel and$\sigma$is the value of filter parameter.

(2) **First-order statistic (FOS) features**

1. **Intensity-Max (FOS_Max)**
2. **Intensity-Min (FOS_Min)**
3. **Intensity-Median (FOS_Median)**
4. **Intensity-Mean (FOS_Mean)**
5. **Intensity-Range (FOS_Range)**
6. **Intensity-Mean absolute deviation (FOS_MAD)**
7. **Intensity-Root mean square (FOS_RMS):**
8. **Intensity-Energy (FOS_Energy)**
9. **Histogram-Variance (FOS_Variance)**
10. **Histogram-Skewness (FOS_Skewness)**
11. **Histogram-Kurtosis (FOS_Kurtosis)**
12. **Histogram-Entropy (FOS_Entropy)**
13. **Histogram-** **Standard deviation (FOS_Std)**

(3) **Gray-Level Co-Occurrence Matrix (GLCM) based features**

GLCM based features were second-order statistical texture features, which are defined as a matrix $M(i,j;\delta,\theta)$ to indicate the relative frequency with intensity values of pixels (*i* and *j*) at the distance of$\delta$in direction$\theta$. We set $\delta=1$ and$\theta=0,45,90,135$. The number of discrete intensity levels in the images was set as 64.

1. **Energy (GLCM_energy)**
2. **Contrast (GLCM_contrast)**
3. **Entropy (GLCM_entropy)**
4. **Homogeneity 1 (GLCM_homogeneity1)**
5. **Homogeneity 2 (GLCM_homogeneity2)**
6. **Correlation 1 (GLCM_correlation1)**
7. **Correlation 2 (GLCM_correlation2)**
8. **Sum of Squares: Variance (GLCM_variance)**
9. **Sum Average (GLCM_saverage)**
10. **Sum Entropy (GLCM_sentropy)**
11. **Dissimilarity (GLCM_dissimilarity)**
12. **Autocorrelation (GLCM_autocorrelation)**
13. **Cluster Prominence (GLCM_cprominence)**
14. **Cluster Shade (GLCM_cshade)**
15. **Difference Entropy (GLCM_dentropy)**
16. **Difference Variance (GLCM_dvariance)**
17. **Maximum Probability (GLCM_mprobability)**
18. **Sum variance (GLCM_svariance)**
19. **Informational measure of correlation 1 (GLCM_IMC1)**
20. **Informational measure of correlation 2 (GLCM_IMC2)**
21. **Inverse Difference Moment Normalized (GLCM_IDMN)**
22. **Inverse Difference Normalized (GLCM_IDN)**

Reference

**1.** Aerts HJ, Velazquez ER, Leijenaar RT, et al. Decoding tumour phenotype by noninvasive imaging using a quantitative radiomics approach. *Nature communications.* 2014;5:4006.

**2.** Fan L, Fang M, Li Z, et al. Radiomics signature: a biomarker for the preoperative discrimination of lung invasive adenocarcinoma manifesting as a ground-glass nodule. *European radiology.* 2019;29(2):889-897.

**Appendix 4: Feature selection method and radiomic signature construction**

We success built a model for predicting non-responders on only the primary cohort, and the validation cohort was only for testing the generalization performance of this model. Before modeling, a feature selection program consisting of 3 steps was executed on the primary cohort. Firstly, the Wilcoxon rank-sum test was performed for every feature, between the TRG 3 and TRG 0–2 groups, as a rough identification of features with P ≤ 0.1 to be used in further processing. Secondly, the Spearman correlation coefficient was calculated between any 2 features, and the feature with the bigger Wilcoxon rank-sum test P value was excluded when the absolute value of the correlation coefficient exceeded 0.9. Thirdly, the least absolute shrinkage and selection operator (LASSO) method was applied to select the most predictive features. To avoid over-fitting, the best LASSO regularization parameter “lambda” was determined by a 10-fold cross-validation. Features within 1 standard error of the minimum criterion were selected for modeling. Then, a multivariate logistic regression model was built based on the selected features. Summation of the selected features multiplied by corresponding coefficients was calculated for each patient as an MPR signature, which can be mathematically represented as follows:

$radiomic signature=\sum_{i=1}^{n} C_{i}*X_{i}+b$, (1)

where $Y$ is the probability of TRG 3 predicted by this model, $b$ the intercept, $X_{i}$ the $i^{th}$selected feature, and $C_{i}$ the coefficient of the $i^{th}$ selected feature. Then, the model can written as:

$Y=\frac{1}{1+e^{-\left( \sum_{i=1}^{n} C_{i}*X_{i}+b \right)}}.$ (2)

All steps of the feature selection and radiomic signature construction were performed with R software using the “glmnet” and “glm2”packages.

**Tables**

**Appendix Table A1 The technical MRI parameters of the scanning sequences.**

| Scanner | Sequence | b value (s/mm^2^) | TR  (ms) | TE  (ms) | Flip Angle | Matrix | Pixel Spacing (mm^2^) | Slice Thickness (mm) | Slice Gap (mm) |
| --- | --- | --- | --- | --- | --- | --- | --- | --- | --- |
| GE 1.5T  (Optima MR360) | DWI | 0/800 | 4600 | 85 | 90^。^ | 256 × 256 | 1.56 × 1.56 | 5 | 6 |
|  | T1w | - | 680 | 13 | 90^。^ | 512 × 512 | 0.55 × 0.55 | 5 | 6 |
|  | CE-T1w | - | 680 | 13 | 90^。^ | 512 × 512 | 0.55 × 0.55 | 5 | 6 |
|  | T2w | - | 4300 | 120 | 90^。^ | 512 × 512 | 0.55 × 0.55 | 5 | 6 |

*DWI* diffusion-weighted imaging, *T1w* T1-weighted, *CE-T1w* contrast-enhanced T1-weighted, *T2w* T2-weighted.

**Appendix Table A2 Summary of radiomic features extracted in the present study.**

| Original features | LoG filter features |
| --- | --- |
| FOS_name | LoG_σ-FOS_name |
| GLCM_name_θ | LoG_σ-GLCM_name_θ |

σ represents the filter value applied, which could be 1.0, 1.5, 2.0, 2.5 and 3.0.

θ represents the considered direction, which could be 0°, 45°, 90°, and 135°.

*FOS* First-order statistic，*GLCM* gray-level co-occurrence matrix, *LoG* Laplacian of Gaussian.

**Appendix Table A3 Clinical characteristics of patients in the primary and validation cohorts.**

| Characteristic | Primary cohort | Validation cohort | P |
| --- | --- | --- | --- |
| TRG  TRG 3  TRG 0–2 |  |  | 1 |
|  | 39 | 13 |  |
|  | 279 | 94 |  |
| Age(years) | 53.53 ± 12.62 | 54.48 ± 10.89 | 0.489 |
| Gender |  |  | 0.389 |
| Male | 222 | 80 |  |
| Female | 96 | 27 |  |
| CEA(ng/ml) |  |  | 0.569 |
| Positive | 125 | 46 |  |
| Negative | 193 | 61 |  |
| cT-stage |  |  | 0.361 |
| T2 | 25 | 4 |  |
| T3 | 232 | 82 |  |
| T4 | 61 | 21 |  |
| cN-stage |  |  | 0.238 |
| N0 | 68 | 25 |  |
| N1 | 126 | 50 |  |
| N2 | 124 | 32 |  |

Continuous data are given as mean ± standard deviation.

P values for categorical variables, such as sex and CEA, were from Fisher’s exact test analysis.

P values for categorical variables, such as cT-stage and cN-stage, were from Pearson’s chi-square test analysis.

P values for continuous variables were from independent samples t-test analysis.

*TRG* tumor regression grade, *cT-stage* clinical T stage, *cN-stage* clinical N stage.

**Appendix Table A4 Table of AUC, accuracy, sensitivity and specificity of MPR and CMPR models according to the Youden index cutoff.**

|  | MPR  Youden index cutoff=-1.749 | | CMPR  Youden index cutoff=-1.515 | |
| --- | --- | --- | --- | --- |
|  | **Primary cohort** | **Validation cohort** | **Primary cohort** | **Validation cohort** |
| AUC (95% CI) | 0.822 (0.752–0.891) | 0.773 (0.608–0.937) | 0.843 (0.779–0.902) | 0.744 (0.585–0.909) |
| Accuracy (95% CI) | 77.04% (72.50%–81.67%) | 76.64% (68.62%–84.55%) | 82.08% (77.86%–86.25%) | 76.64% (68.78%–84.61%) |
| Sensitivity (95% CI) | 74.36% (60.68%–87.83%) | 61.54% (34.39%–88.82%) | 74.36% (60.56%–88.37%) | 53.85% (25.77%–82.00%) |
| Specificity (95% CI) | 77.41% (72.57%–82.40%) | 78.72% (70.47%–86.85%) | 83.15% (78.74%–87.49%) | 79.79% (71.58%–88.08%) |

*AUC*, area under the receiver operating characteristic curve; *MPR*, multi-parametric MRI-based radiomic model; *CMPR*, combined MPR signature and age model; *CI*, confidence interval.

**Appendix Table A5 Risk Factors for non-response to** **neoadjuvant therapy in** **locally advanced rectal cancer.**

| **Intercept and Variable** | **modality** | **MPR model** | | |
| --- | --- | --- | --- | --- |
|  |  | **coefficient** | **OR (95% CI)** | **p** |
| Intercept | - | -11.6970 | - | 0.0004 |
| LoG_1.5-GLCM_correlation1_0 | T1w | -1.7043 | 0.56 (0.23-1.35) | 0.1947 |
| LoG_1-GLCM_saverage_90 | CE-T1w | 2.6541 | 3.03 (1.53-5.97) | 0.0014 |
| LoG_2-GLCM_mprobability _45 | ADC | -3.4363 | 0.61 (0.23-1.57) | 0.3026 |
| LoG_2-GLCM_mprobability_0 | ADC | -0.8859 | 0.82 (0.31-2.19) | 0.6959 |
| LoG_3-GLCM_correlation1_90 | T1w | 1.4344 | 1.53 (0.54-4.39) | 0.4254 |
| GLCM_homogeneity2_90 | ADC | 0.0015 | 1.00 (0.32-3.14) | 0.9994 |
| LoG_1-GLCM_correlation1_90 | ADC | 1.1647 | 1.26 (0.76-2.10) | 0.3649 |
| GLCM_dissimilarity_90 | ADC | -1.1269 | 0.70 (0.22-2.28) | 0.5587 |
| LoG_1-GLCM_correlation1_135 | CE-T1w | 1.5984 | 1.76 (1.00-3.09) | 0.0498 |
| GLCM_cshade_45 | ADC | 0.7817 | 1.27 (0.75-2.17) | 0.3789 |
| GLCM_IMC1_0 | CE-T1w | 0.6623 | 1.25 (0.69-2.27) | 0.4578 |
| LoG_3-GLCM_dentropy_90 | T2w | -0.4331 | 0.82 (0.44-1.55) | 0.5489 |
| GLCM_entropy_135 | T1w | 1.7350 | 3.28 (1.41-7.64) | 0.0059 |
| LoG_1-FOS_Median | ADC | 2.0526 | 1.24 (0.76-2.01) | 0.3946 |
| FOS_Skewness | ADC | 0.7852 | 1.34 (0.74-2.43) | 0.3354 |
| LoG_3-FOS_Mean | T1w | 4.3126 | 1.76 (0.99-3.10) | 0.0516 |

*FOS* First-order statistic, *GLCM* gray-level co-occurrence matrix, *LoG* Laplacian of Gaussian, *IMC1* Informational measure of correlation 1. *MRI* magnetic resonance imaging, *MPR* multi-parametric MRI-based radiomic model, *ADC* apparent diffusion coefficient, *T1w* T1-weighted, *CE-T1w* contrast-enhanced T1-weighted, *T2w* T2-weighted, *OR* odds ratio.

**Figures**

**Appendix** **Fig. A1**

**
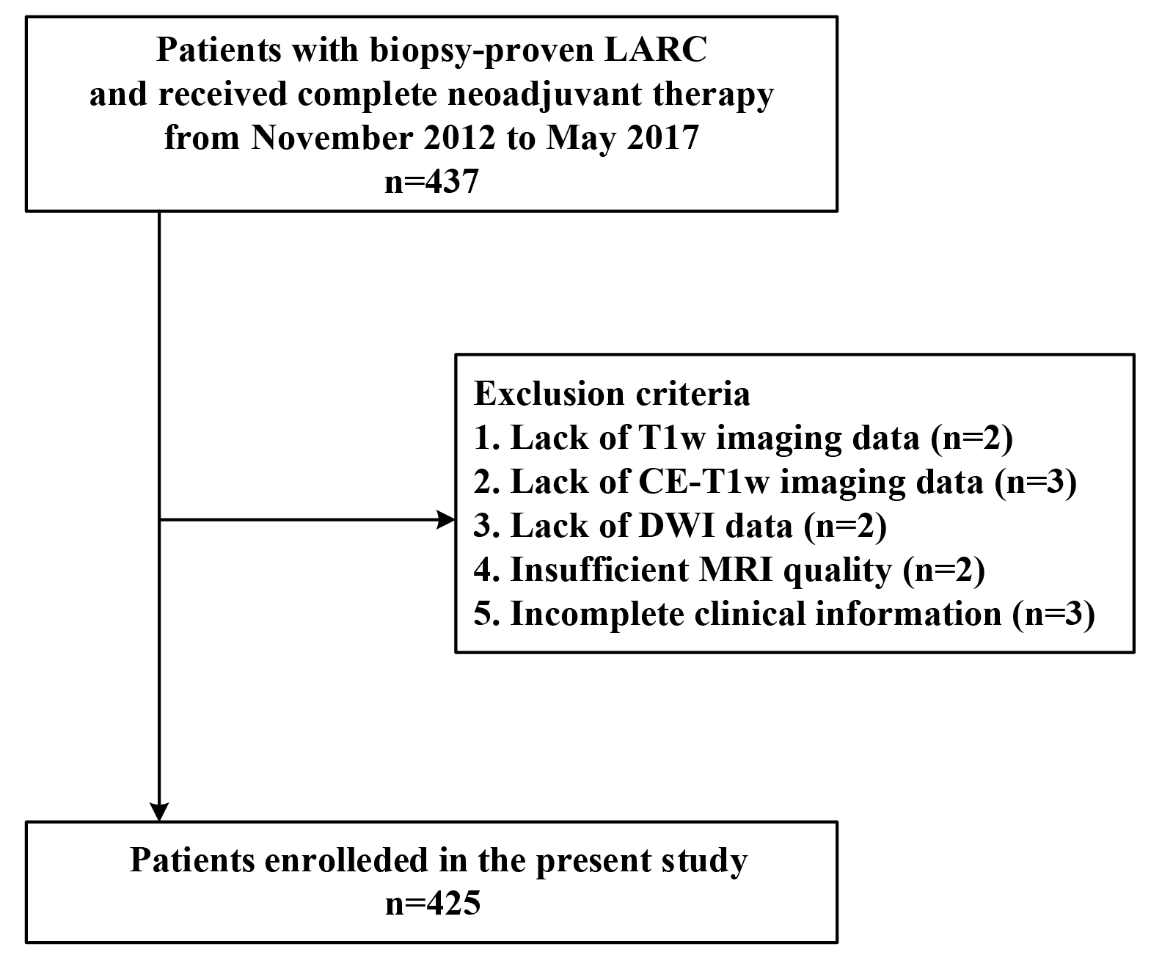
**

**Fig. A1 Recruitment pathway for patients in this study.** *LARC* locally advanced rectal cancer, *T1w* T1-weighted, *CE-T1w* contrast-enhanced T1-weighted, *DWI* diffusion-weighted imaging, *MRI* magnetic resonance imaging.

**Appendix Fig. A2**

**
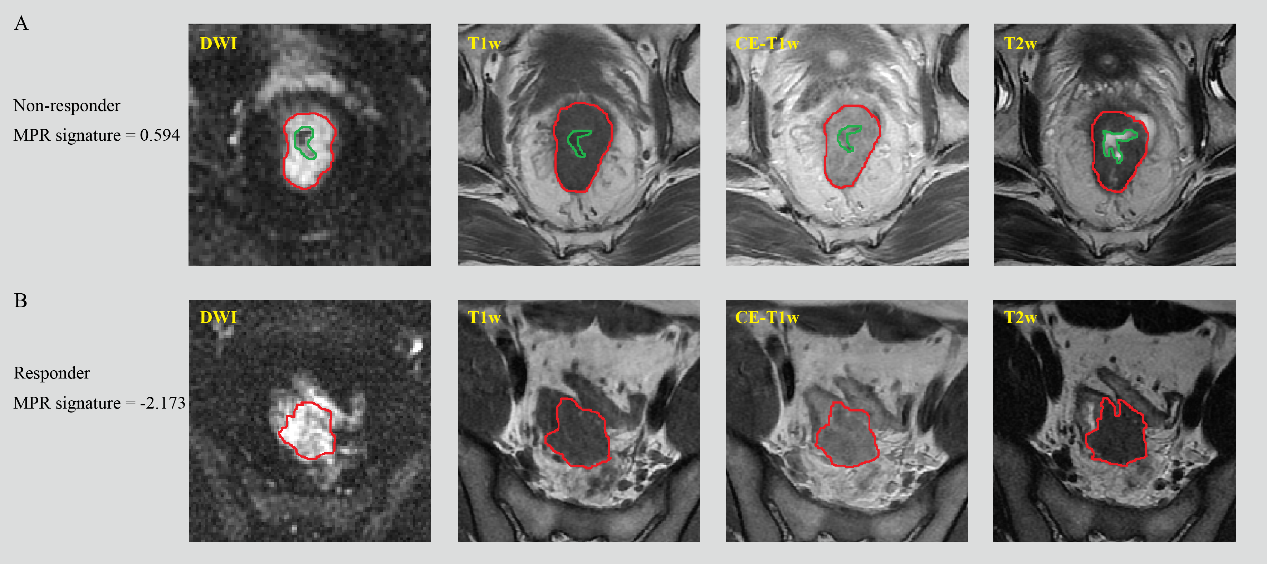
**

**Appendix Fig. A2 Manual segmentation on images of different modalities.** (A) sample images of non-responder; red line indicates an outer margin, green line indicates an inner margin. (B) sample images of responder. *ROI* region of interest**,** *MRI* magnetic resonance imaging, *MPR* multi-parametric MRI-based radiomic**,** *DWI* diffusion-weighted imaging, *T1w* T1-weighted, *CE-T1w* contrast-enhanced T1-weighted, *T2w* T2-weighted.
